# Communication Routes in ARID Domains between Distal Residues in Helix 5 and the DNA-Binding Loops

**DOI:** 10.1371/journal.pcbi.1003744

**Published:** 2014-09-04

**Authors:** Gaetano Invernizzi, Matteo Tiberti, Matteo Lambrughi, Kresten Lindorff-Larsen, Elena Papaleo

**Affiliations:** 1 Structural Biology and NMR Laboratory, Department of Biology, University of Copenhagen, Copenhagen, Denmark; 2 Department of Biotechnology and Biosciences, University of Milano-Bicocca, Milan, Italy; Max Planck Institute for Biophysical Chemistry, Germany

## Abstract

ARID is a DNA-binding domain involved in several transcriptional regulatory processes, including cell-cycle regulation and embryonic development. ARID domains are also targets of the Human Cancer Protein Interaction Network. Little is known about the molecular mechanisms related to conformational changes in the family of ARID domains. Thus, we have examined their structural dynamics to enrich the knowledge on this important family of regulatory proteins. In particular, we used an approach that integrates atomistic simulations and methods inspired by graph theory. To relate these properties to protein function we studied both the free and DNA-bound forms. The interaction with DNA not only stabilizes the conformations of the DNA-binding loops, but also strengthens pre-existing paths in the native ARID ensemble for long-range communication to those loops. Residues in helix 5 are identified as critical mediators for intramolecular communication to the DNA-binding regions. In particular, we identified a distal tyrosine that plays a key role in long-range communication to the DNA-binding loops and that is experimentally known to impair DNA-binding. Mutations at this tyrosine and in other residues of helix 5 are also demonstrated, by our approach, to affect the paths of communication to the DNA-binding loops and alter their native dynamics. Overall, our results are in agreement with a scenario in which ARID domains exist as an ensemble of substates, which are shifted by external perturbation, such as the interaction with DNA. Conformational changes at the DNA-binding loops are transmitted long-range by intramolecular paths, which have their heart in helix 5.

## Author Summary

Regulation of DNA transcription is a key element for the cell to finely regulate its physiological processes. This is acquired by the use of a special class of proteins that bind to DNA and function as transcriptional regulators. ARID domains are responsible for many of these DNA-protein interactions in proteins involved in important physiological functions, including cell-cycle regulation and embryonic development. Nevertheless, the structural effects and the conformational changes induced by DNA-binding on the ARID structure have been poorly characterized so far. Here, we provide the first characterization of long-range effects induced by DNA-binding on ARID domains. In particular, we identified routes of communications from DNA-binding loops to distal residues in helix-5. These routes pre-exist in the free protein and are strengthened by DNA interaction. We also investigated mutations that experimentally impair DNA-binding. Our results show that these mutations perturb wild-type routes of communication, allowing to link their effect on structure and dynamics to function. We have also found a region that might be used for recruitment of biological partners. We predicted the effects induced by mutations at other crucial sites for ARID dynamics. Our results are thus likely to boost the future experimental and structural research on ARID domains.

## Introduction

ARID3A is a member of the ARID (AT-rich interactive domain) family of transcription factors and is also known as “dead ringer-like protein 1” (Dril1), “B-cell regulator of IgH transcription” (Bright) and “E2F-binding protein 1” (E2FBP1). The ARID family is a family of DNA-binding proteins with a wide range of cellular functions and participates in different regulatory processes, including embryonic development, gene expression during cell growth, differentiation and development as well as cell cycle control and chromatin remodeling [1]–[4]. Human ARID3A is also one of the targets of the broad Human Cancer Protein Interaction Network (HCPIN) database, which aims to provide structure-function annotations of key proteins related to cancer diseases and developmental biology [5]. ARID domains have been identified in the genomes of higher eukaryotes and feature a common all-α structural domain of about 100 residues or longer [1].

ARID proteins bind to the major groove in the DNA using a modified helix-turn-helix motif [1], [2]. Human ARID3A belongs, together with ARID3B and 3C, to the third mammalian ARID subfamily, which is generally characterized by a core ARID domain with both additional N- and C-terminal extensions [1], [2]. ARID3A, B and C are the closest paralogs (more than 75% of sequence identity) of the Drosophila “dead ringer” protein Dri [1].

The structures of the free ARID3A [6] and of both the free and DNA-bound Drosophila paralog [7], [8] were recently solved by NMR spectroscopy and X-ray crystallography. Indeed, despite their biological importance ARID domains are still relatively poorly characterized from the structural and dynamical point of view. Considering their importance for DNA interaction and the involvement in a large range of biological functions, ARID domains are suitable targets for Molecular Dynamics (MD) investigations with attention to both dynamic fingerprints and structural communication mechanisms. In addition to providing basic information on the dynamics of ARID proteins such analyses may shed light on the structural effects induced by mutations.

Here, we have studied intramolecular communication in two members of the ARID family, both in the free and DNA-bound states, with particular attention to the effects induced by distal residues on the DNA-binding loops. We used an approach that integrates atomistic, explicit solvent simulations, prediction of the effects induced by mutations on protein stability and methods inspired by graph theory. In particular, we found that the interaction with the DNA strengthens pre-existing paths from distal sites in helix α5 to the DNA-binding loops. In addition to the residues directly involved in the DNA binding, a distal tyrosine (Y119) was identified. This residue affects DNA-binding as attested by experimental mutagenesis and a Electrophoretic Mobility Shift Assay (EMSA) [9]. We here show that Y119 plays a key role in promoting long-range communication to the loops at the interface with DNA. Other residues, mostly in helix α5, are also identified with a key role in intramolecular communication and we show that their mutations can impair the native paths to the DNA-binding regions. The surroundings of Y119 are also predicted as hotspots for protein-protein interaction, suggesting that paths identified in our study may also be an important element to propagate effects long-range from the DNA-binding site to a region for the recruitment of other biological partners, or *vice versa*.

## Results

### The target proteins

**Figure 1:**
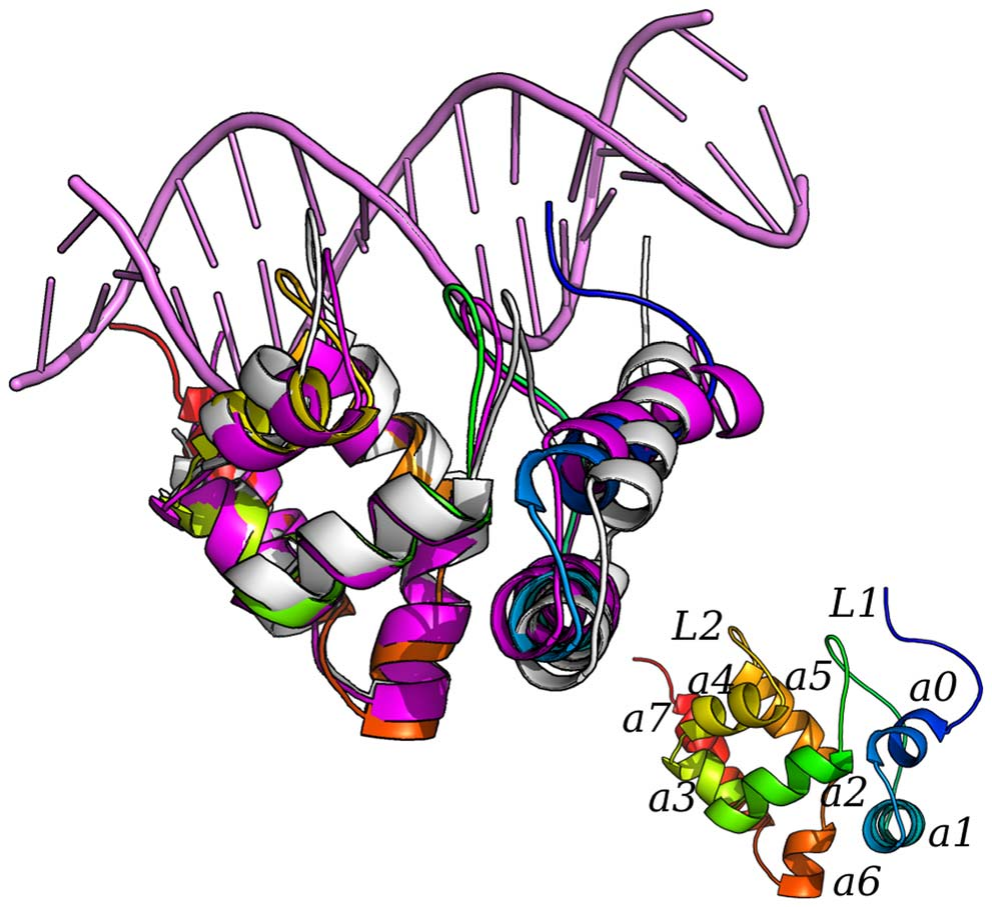
Target proteins and structural features. The 3D structures of ARID3A_FREE_ (PDB entry 2KK0), Dri_FREE_ (PDB entry 1C20) and Dri-DNA (PDB entry 1KQQ) are shown as yellow, light grey and magenta cartoons, respectively. In the figure at the bottom on the left the structure is colored with different shade of colors from the N- (blue) to the C-terminal extremity (red) and the secondary structural elements are labelled according to ref. [6]. In particular, α0–α7 (residues 231–234, 239–254, 272–282, 285–291, 294–300, 310–321, 324–329, 335–346 in ARID3A or according to the PDB entry 2KK0 numbering 25–28, 33–48, 66–76, 79–85, 88–94, 104–115, 118–123, 129–140) and a very short β-harpin consisting of antiparallel strands β1 and β2 (residues 264–265 and 268–269 in ARID3A or according to the PDB entry 2KK0 numbering 58–59 and 62–63) are shown.

A short description of the target proteins is reported in the following (**Figure 1**). The ARID domain of ARID3A and Dri consists of eight α-helices (α0–α7) and a very short β-harpin. We here refer to the numbering of ARID3A NMR structure (2KK0) in the Protein Data Bank (PDB) for sake of clarity. A sequence alignment is also provided with the corresponding numbering in ARID3A and Dri, (**Figure S1**). Loop L2 and the β-harpin Loop L1 of Dri interact with the DNA major groove and regions outside the major groove, respectively [1], [7]. These residues are also conserved in ARID3A, suggesting a common binding mode to the DNA [6]. The results are also in agreement with the recent experimental finding that L1 of human JARID1B ARID domain is crucial for DNA binding [10]. Therefore, we here used the X-ray structure of Dri bound to DNA as a reference for the DNA-bound ARID domains.

### Evaluation of the simulated ensembles and comparison to the experimental data

The MD simulations collected in this study are summarized in **Table 1**. At first, we carried out ten independent simulations for ARID3A_FREE_ and DRI_FREE_ of 100 ns each to assess the reproducibility of the results. One of the DRI_FREE_ simulations was also extended up to one microsecond (Dri_FREE-1 µs_). The Dri DNA-bound conformation (PDB entry 1KQQ) was employed as starting structure for four 100-ns MD replicates of Dri in complex with DNA (Dri_DNA_). All the simulations have been performed with CHARMM22*, a new generation force field, which was validated against NMR data [11].

**Table 1:** Summary of the MD simulations.

Simulated system	Starting Structure	Duration of each replicate (ns)	# replicates
Dri_FREE_	Dri NMR structure (1C20 –conformer 1)	100	6
Dri_FREE-1 µs_	Dri NMR structure (1C20 –conformer 1)	1000	1
Dri_DNA_	Dri in complex with DNA NMR structure (1KQQ)	100	4
Dri_FREE-Q101N_	Dri NMR structure (1C20 –conformer 1) – *in-silico* mutation Q101N	100	2
Dri_FREE-Q101A_	Dri NMR structure (1C20 –conformer 1) – *in-silico* mutation Q101N	100	2
Dri_FREE-Y109A_	Dri NMR structure (1C20 –conformer 1) – *in-silico* mutation Q101N	100	2
Dri_DNA-Q101N_	Dri in complex with DNA NMR structure (1KQQ) - *in-silico* mutation Q101N	100	2
Dri_DNA-Q101A_	Dri in complex with DNA NMR structure (1KQQ) - *in-silico* mutation Q101A	100	2
Dri_DNA-Y019A_	Dri in complex with DNA NMR structure (1KQQ) - *in-silico* mutation Y109A	100	2
ARID3A_FREE_	Human ARID3A NMR structure (2KK0 – conformer 1)	100	4
ARID3A_FREE_ unfolding simulation at 500 K	Human ARID3A NMR structure (2KK0 – conformer 1)	100	1
Dri_FREE_ unfolding simulation at 500 K	Dri NMR structure (1C20 –conformer 1)	100	1
ARID3A_Q111N_	Human ARID3A NMR structure (2KK0 – conformer 1) – *in-silico* mutation Q111N	100	2
ARID3A_Q111A_	Human ARID3A NMR structure (2KK0 – conformer 1) – *in-silico* mutation Q111A	100	2
ARID3A_Y119A_	Human ARID3A NMR structure (2KK0 – conformer 1) – *in-silico* mutation Y119A	100	2

All the simulations were performed with CHARMM22* force field.

To verify that our simulations do not encounter issues related to force-field deterioration or low stability of the sampled structures, chemical shift predictions of backbone and Cβ atoms were calculated by PPM [12] and compared to the experimental values (**Table 2** **, Figure S2**). Indeed, we need to sample conformations that do not deviate from the experimental data to avoid artifact arising from the analyses of the MD ensemble. The average root mean square deviation (rmsd) between experimental and predicted chemical shifts was then calculated for the MD ensemble [12] and compared to the results obtained for the starting experimental structures (**Table 2**). The data obtained for all the five chemical shift classes show that the rmsd values are within or near the expected deviations recorded for the protein test sets and substantially lower than the rmsd values calculated on the starting structures (PDB entries 2KK0 and 1C20). Moreover, the time-evolution of the backbone and Cβ chemical shifts was evaluated for Dri_FREE-1 µs_ (**Figure S2**). The one-µs trajectory has rmsd values comparable to the single 100-ns replicates (**Table 2**). Further, the evolution of the chemical shift rmsd over the simulation time (**Figure S2**) reveals a stabilization or improvement of those values after the first 100 ns of simulation. Overall, these results indicate that the trajectories analyzed are stable and with rmsd values substantially lower than those calculated from the PDB starting structure (green lines in **Figure S2**) when at least 100 ns of MD ensemble are collected. Moreover, a ten-fold increase of the simulation time does not lead to a concrete improvement of the rmsd values suggesting that 100 ns is an adequate timeframe for our analysis.

**Table 2:** Prediction of backbone chemical shifts by PPM in ARID3A_FREE_ and Dri_FREE_ simulations.

MD	PPM-Cα	PPM-Cβ	PPM-C	PPM-HN	PPM-N
**PPM-test set** a	1.06	1.23	1.32	0.53	2.91
**ARID3Afree-r1**	1.17	0.99	1.29	0.54	2.87
**ARID3Afree-r2**	1.23	1.01	1.28	0.53	2.97
**ARID3Afree-r3**	1.20	1.01	1.23	0.55	2.86
**ARID3Afree-r4**	1.23	0.93	1.27	0.55	2.93
**Dri_FREE_**	1.20	1.22	1.25	0.56	3.12
**Drifree-r2**	1.46	1.34	1.35	0.56	3.77
**Drifree-r3**	1.41	1.38	1.28	0.54	3.51
**Drifree-r4**	1.22	1.31	1.26	0.59	3.34
**Drifree-r5**	1.28	1.27	1.31	0.54	3.23
**Drifree-r6**	1.37	1.43	1.31	0.57	3.3
**Drifree-1 µs**	1.33	1.17	1.3	0.52	3.31
**2KK0**	1.36	1.36	1.42	0.48	2.76
**1C20**	1.87	2.20	1.56	0.75	2.83

Rmsd between calculated and experimental backbone chemical shifts are reported. In particular, chemical shifts were calculated for C, N, HN, Cβ, and Cα atoms on structures from the equilibrium trajectories collected every 100 ps.

a For comparison, the deviations between experimental and PPM-predicted chemical shifts for protein test sets are provided as reported in ref. [12].

We can thus post-process the MD ensembles by methods inspired by graph theory to derive paths of communications, other important properties of the network (as for example hub residues) and to investigate how (and if) those paths are affected by the interaction with DNA and by mutations. The availability of one µs simulation for Dri_FREE_ also allowed us to assess the influence, on the PSN description, of simulating the system over a longer timescale.

### Correlated motions in the MD ensemble

**Figure 2:**
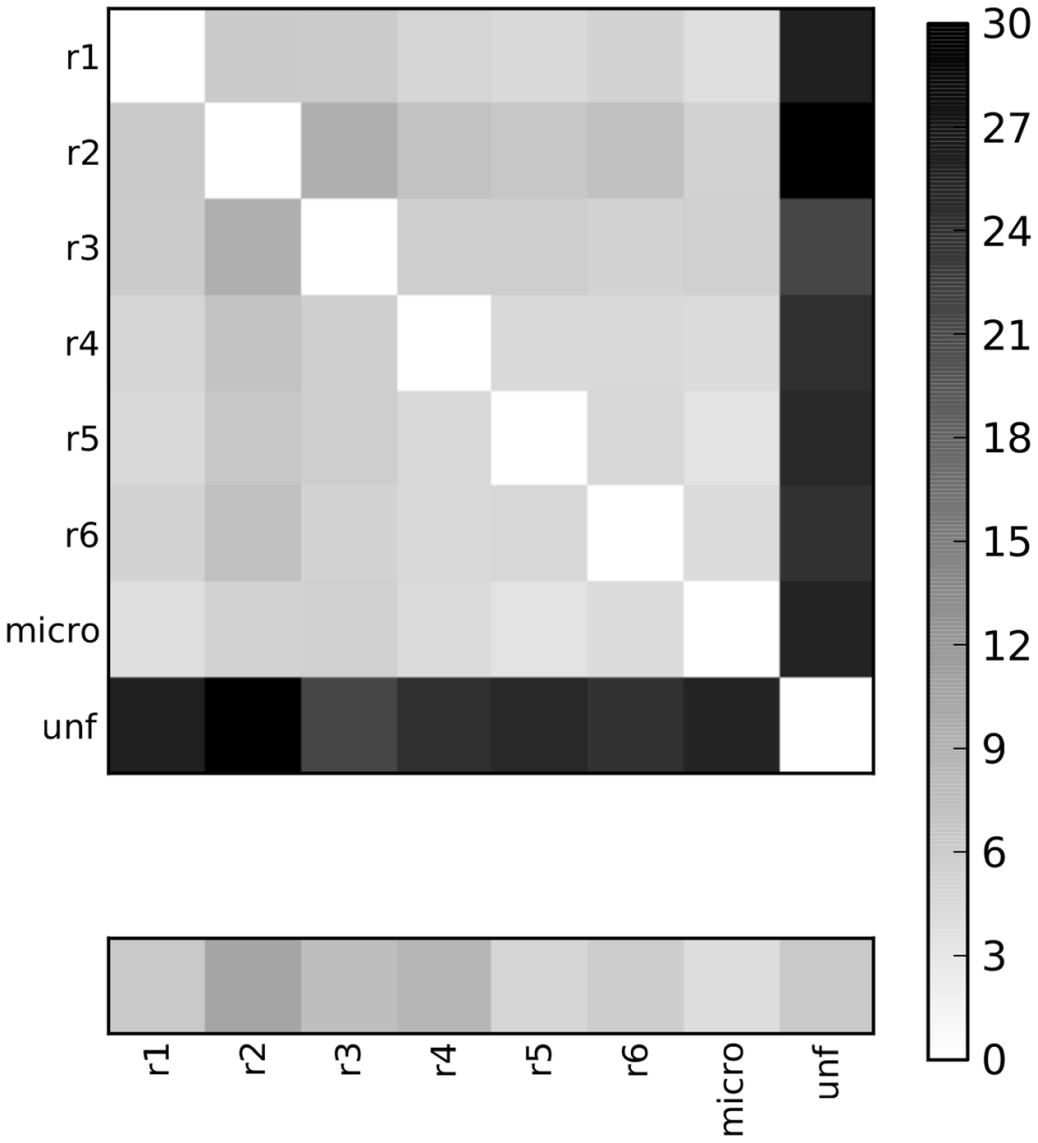
Comparison of LMI matrices describing correlated motions. The Frobenius norms between average LMI matrices calculated from different replicates of the same target protein are reported. An average LMI matrix achieved from an unfolding simulation at 500K of the same protein is used as a control. The example of Dri_FREE_ simulations is reported for sake of clarity. Similar results with even lower Frobenius norms were obtained for ARID3A_FREE_ replicates (average Frobenius norm of 5). Average LMI were calculated with five-ns time-windows, as explained in Materials and Methods. The box on the bottom shows the values of Frobenius when the two halves of each replicate are compared to use as a baseline in the comparison.

We here employed a method inspired by graph-theory, the so-called Protein Structure Network (PSN)-MD approach [13]–[15] to detect the paths of communication in the MD ensembles of ARID3A and Dri. This method is based on the observation that structural effects can be transmitted at distal sites through communication paths involving side-chain contacts between residues that feature correlated motions [13]–[15]. The PSN calculation is thus integrated to metrics that estimate coupled motions from the MD ensemble. We here employed Linear Mutual Information (LMI) [16] as a metrics of correlated motions. To assess the consistency of the results, we compared LMI matrices from each independent replicate. In particular, LMI matrices were calculated as average matrices over time-windows of five ns (**Figure S3**). The correlated motions are similarly and consistently described. The maximum differences observed for the LMI matrices are always below 0.35 and are generally restricted to the N- and C-terminal residues. To better quantify the differences among LMI matrices of the same system, we also calculated the Frobenius norm between them (**Figure 2**). If the description of the correlated motions is similar in all the replicates, we expect low values of Frobenius norm when they are compared pairwise. The wild-type LMI matrices of ARID3_AFREE_ and Dri_FREE_ simulations were also compared to the LMI matrices calculated from 100-ns unfolding simulations at 500 K of the same proteins. Indeed, if the different LMI matrices of the wild-type protein are really consistent in describing the correlated motions of the protein, the Frobenius norm obtained from their pairwise comparison should be at least lower than the ones achieved when each of the wild-type LMI matrices is compared to the LMI matrix from the unfolding simulation, in which the native structure is not preserved. All the replicates of the same system feature lower Frobenius norm values when compared each other than when compared to the corresponding unfolding simulation (illustrated in **Figure 2** for Dri_FREE_) and they are always within the range of values of the LMI Frobenius norm calculated between the two halves of the same target trajectory (average value of 6.80, bottom insert in **Figure 2**). Indeed, as expected, the LMI matrix from the unfolding simulation largely deviates from the LMI of the folded proteins (average Frobenius norm of 22.13).

In summary, LMI matrices calculated from the different replicates of the same system and averaged over different time-windows consistently describe the same pattern of correlated motions and can thus be used, coupled to network analysis, to disclose paths of long-range communication in the MD ensembles.

### PSN analysis and definition of the Interaction strength (I) cutoff

We then calculated the PSN from each MD replicate. In this network the residues are the nodes of the graph and are connected by edges weighted according to a defined Interaction strength (*I*) value [17]. In the PSNs, the calculated edges are retained only if their *I* is greater than a defined cut-off value (minimum Interaction Strength, *I_min_*). Generally, a PSN at the so-called *I_crit_* value is calculated for further analysis, where the *I_cri_*_t_ is the *I_min_* corresponding to the main transition in the size of the largest cluster (cluster 1) of the network [17].

**Figure 3:**
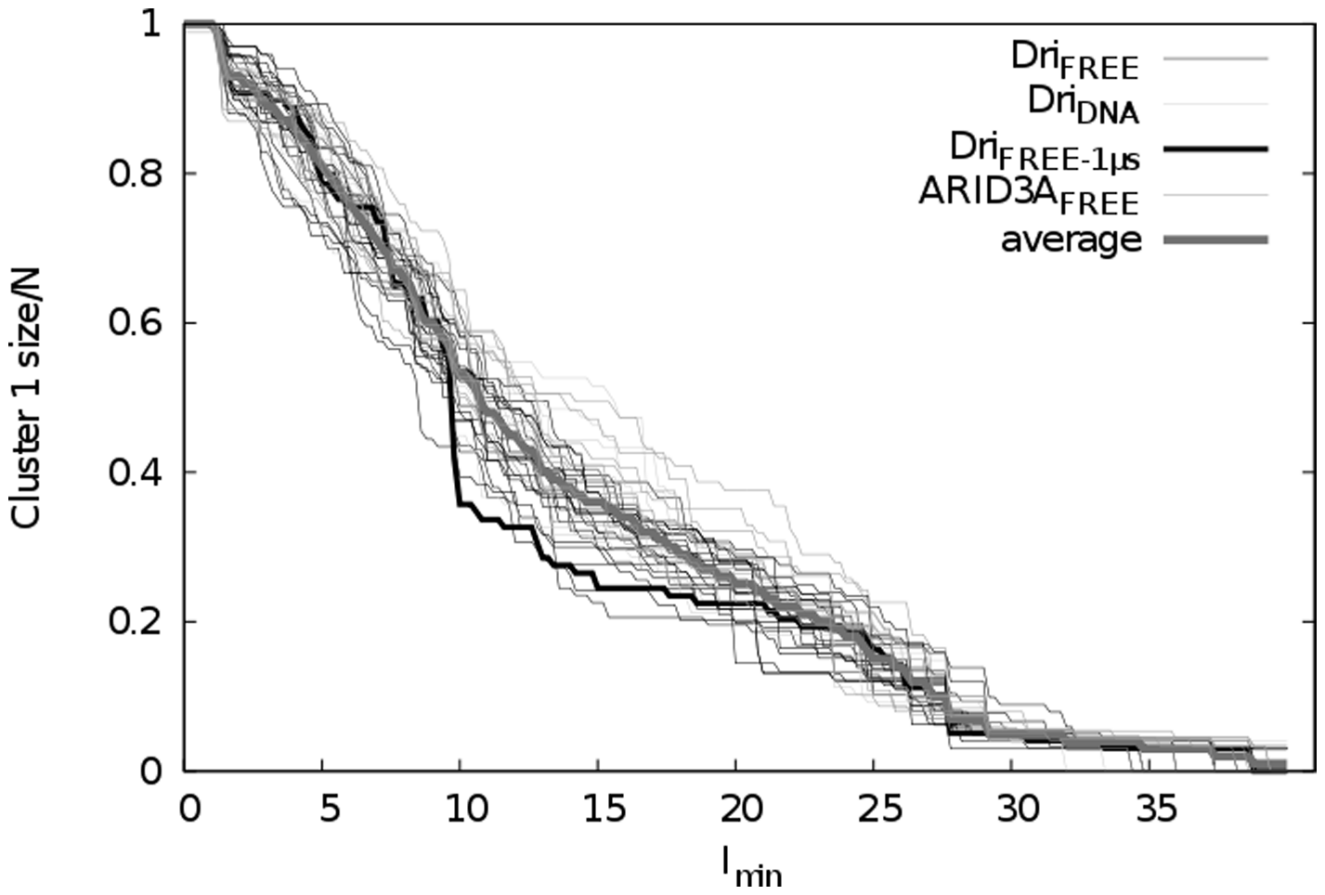
Evolution of the size of the largest cluster (cluster 1) as a function of the *I_min_* values in Dri and ARID3A simulations. The *I_crit_* value for the PSN analysis was calculated for each of the simulations collected in this study, included wild-type, mutant variants and DNA-bound structures. The analyses point out the consistency of this parameter within proteins sharing the same fold but different primary sequences, as well as in their DNA-bound and DNA-unbound state, as also previously pointed out for similar analysis on single PDB structures [17].

Both to define the *I_crit_* value for the analysis and to verify the congruency of the results from our simulations, we calculated the evolution of the size of cluster 1 as a function of different *I_min_* values. We thus calculated several PSNs for each replicate, varying the *I_min_* value from 0 to 40 by steps of 0.2 (**Figure 3**). Independently of the system (ARID3A or Dri), presence of mutations, DNA and differences in the timescales, all the MD ensembles feature similar profiles with a first very sharp transition in a narrow range of *I_min_* values. It was previously observed that in PSNs of experimental protein structures collected from the PDB, this transition occurs in the same range of *I_min_* for proteins of different size and fold [17]. Our simulations confirm this finding also for protein structures simulated within a classical force-field description, even if the I*_crit_* (∼7 in our study) is shifted to slightly larger values than what observed in the previous study on single PDB structures [17]. We can thus employ a common I*_crit_* value for all the systems and we here shown the robustness of PSN *I_crit_* even in MD ensembles of not identical proteins.

### Hub residues in ARID domains in their DNA-unbound and -bound states

**Figure 4:**
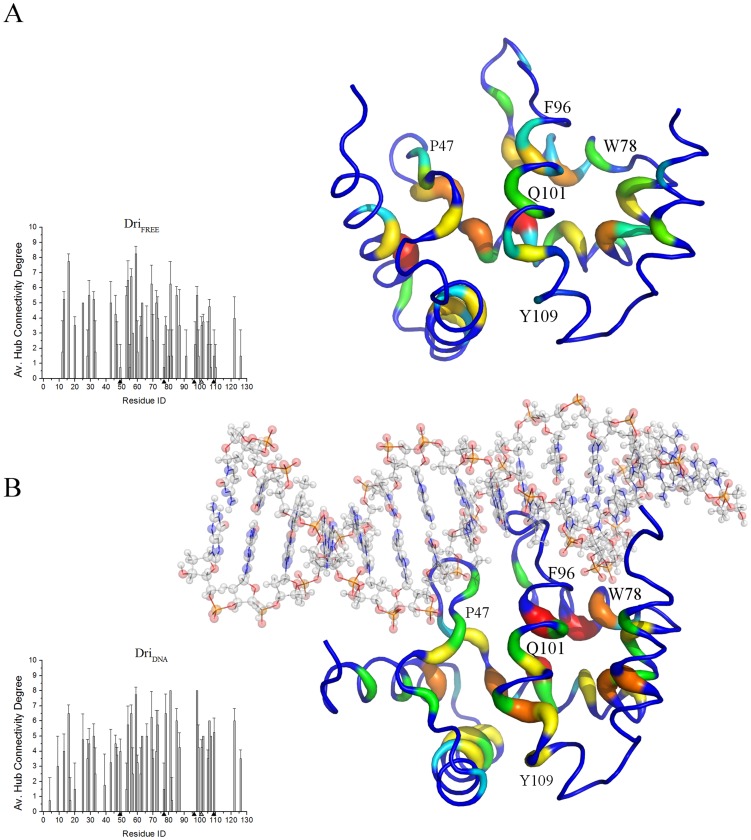
Hub residues and their location on the 3D structure of ARID domains. The connectivity degree for each PSN hub of Dri_FREE_ (A) and Dri_DNA_ (B) simulations are shown as a function of the protein residue in the left panels. Since in a PSN a hub is defined as a residue connected by at least three edges, all the residues with node degree lower than three are set at zero. Black triangles indicate the position of the residues experimentally known to impair DNA-binding [9], whereas a gray triangle indicates Q101/Q111 position. The right panels show the location of these hubs on a reference 3D structure of the corresponding target protein. The structure is depicted as ribbon with rainbow shade of colors according to the node degree. The corresponding figures for ARID3A_FREE_ and 1 µs Dri_FREE_ simulations are shown in Figure S4. The residue numbering is referred to Dri in the figure where P47, W78, F96, Q101 and Y109 correspond to P57, W88, F106, Q111 and Y119 in ARID3A.

Hubs of a PSN are highly connected residues in the network, i.e. nodes connected by more than three edges. They can play a role in protein structural stability, function or allow a proper flux of information to distal sites [17]–[20]. We calculated the PSN hubs for free and DNA-bound ARID domains (**Figure 4** **and S4**). In particular, we calculated the node degree (i.e. the number of edges in which the hub is involved) of each hub for each simulation. Overall, both the hub localization on the structure and their connectivity degree in the network are very similar for the different replicates of the same system even if the PSN is calculated on a longer timescale (i.e. one µs).

Interaction with DNA promotes higher connections not only in the DNA-binding loops, but also for the nodes that are not in direct contact with the DNA (**Figure 4** **and S4**). In particular, the connectivity degree of each hub and the hub number itself increase in the central helix α5 of the ARID domain upon DNA interaction. These are sites that may play a role in long-range communication to distal sites, as detailed in the next Sections.

Hubs in ARID3A_FREE_ and Dri_FREE_ are generally placed at identical positions and most of them are also strictly conserved in terms of primary sequence, enforcing the notion of common dynamic patterns in these two proteins.

Y119 is conserved as a hub residue in most of ARID3A_FREE_ and Dri simulations independently of the presence or absence of DNA, but DNA interaction increases its connectivity degree (**Figure 4** **, S4**). The Y119 in ARID3A (Y109 in Dri) is known to affect DNA binding capability when mutated to alanine [9] even if according to the 3D structure Y119 is not in direct contact with the DNA molecule. It is indeed placed at the C-terminal region of α5 more than 20 Å of distance from the DNA binding site (**Figure S5**). In the same study, the authors identified, by alanine scanning mutagenesis, three other residues that are crucial for DNA binding: P57, W88 and F106 (**Figure S5**, P47, W78 and F96 in Dri). F106/F96 has a minor interest for our work since it is in direct contact with the DNA and is not conserved in all the ARID family members, as attested by a low conservation score of this position in a multiple sequence alignment of 100 sequences homologous to ARID3A by CCRXP server [21]. Both P57/P47 and W88/W78 hub-properties are also modulated by the DNA interaction, as observed for Y119 (**Figure 4** **, S4**). Another interesting hub of α5 is Q111/Q101. It is placed in a position suitable as “mediator” for communication paths and it is a hub residue with higher connectivity upon DNA interaction (**Figure S4**).

We then evaluated by using Fold-X [22] the effects that the experimentally investigated mutations [9], as well as R109A and Q111A or Q111N mutations (see below), may have on the structural stability. All these mutations (Y119A, F106A, R109A, Q111A and Q111N) have only modest effects on protein stability (average ΔΔG values between −0.2 and 0.6 kcal/mol) with the exception of W88A and P57A mutations that have more destabilizing effects (average ΔΔG values of 4.4 and 2.5 kcal/mol, respectively). These two residues (P57 and W88) are also conserved in the multiple sequence alignment of ARID homologs carried out by the CCRXP server [21], with conservation scores of 0.889 and 0.850, respectively.

In our MD simulations, most of the experimental mutation sites (P57, Y119 and W88) [9] act as hubs with or without the DNA and their connectivity within the graph is modulated by the DNA. This observation alone might suggest a central role exerted by these residues that are not in direct contact with the DNA in mediating long-range communication to the DNA-binding interface. Nevertheless, in the case of P57 and W88, the alanine mutations by Fold-X are predicted to remarkably affect protein stability of the ARID domains. In this scenario we can conclude that the effects observed in the experiments upon P57A and W88A mutations are more likely to be related to a destabilization of the protein fold rather than due to distal communication to the L1 and L2 DNA-binding loops. P57 is indeed a residue with a structural role devoted to maintain the local conformation of the L1 β-hairpin and the correct position of K61 for DNA interaction [7]. Y119 instead appears as an important mediator for distal communication and its mutation should not compromise structural stability of the ARID domains.

### Paths of long-range communication to L1 and L2 loops

To better investigate the long-range communication from distal sites of ARID domains to the DNA-binding loops L1 and L2, we then employed a PSN/LMI approach [15], [23]–[24]. In particular, we calculated the shortest paths of long-range communication from each protein residue to the DNA-binding loops L1 and L2. Indeed, the shortest paths of communication are likely to be the paths that more efficiently transmit a “signal” over long distances within the protein structure [20]. The paths were then ranked according to their probability of occurrence and length. The shortest paths with highest occurrence probability that connected two end-residues by a series of non-covalent interactions with highly correlated motions were selected. Particular attention was devoted to the pairs of residues connected by a path with a probability of occurrence higher than 15% to discard paths that are too poorly populated in the conformational ensemble and may thus increase the noise of the analysis. Moreover, to focus our attention on long-range communication, only the paths of length greater than three were analyzed in details.

**Figure 5:**
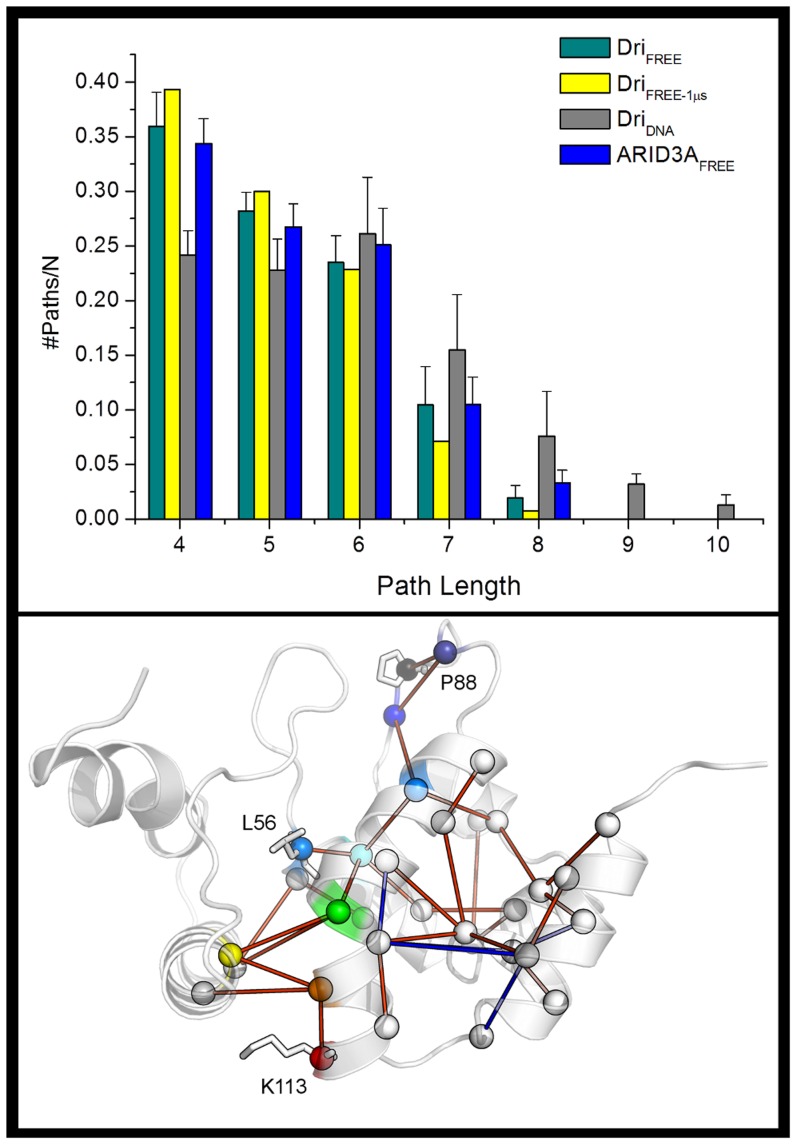
Long-range paths are promoted by DNA interaction in Dri simulations. **Left panel**) Distribution of the shortest paths of communication identified by the PSN-LMI approach in the different simulations employed in this study. Average values from the different replicates of the same system are shown. **Right panel**) Differences among the paths of length higher than 8 residues (starting and final residues included) in Dri_DNA_ and Dri_FREE_ simulations. The paths that are present only in Dri_DNA_ or Dri_FREE_ simulations are shown as red and blue cylinders, respectively. Nodes of the paths are shown as spheres centered on the Cα atom. The nodes belonging to the paths from K113 to both L56 at the base of L1 (K113→Y109→L32→L106→F28→L56) and L87-I91-P88 in L2 (K113→Y109→L32→L106→L59→L98→L87→I91→P88, Dri numbering) are highlighted by different shades of colors from red to blue/black. K113, L56 and P88 are shown as sticks.

Comparing the Dri_FREE_ and Dri_DNA_ simulations (dark green vs. gray histograms in **Figure 5** **, upper panel**), we noticed that long-range paths (from 7 to 10 residues in length) are increased by the DNA interaction, whereas the shorter-range paths (4–5 residues) are decreased. The presence of DNA not only promotes longer paths of communications to L1 and L2 (**Figure 5** **, lower panel**). Indeed, if we used the same PSN/LMI approach to calculate the shortest paths from each residue to other protein sites, with the exception of the DNA binding loops, we observed a 28% decrease of long-range paths directed to sites different from the DNA-binding region. The two results together suggest that DNA promotes a well-channeled long-range communication from specific distal sites to the L1 and L2 binding loops and weakens other communication routes of the unbound protein.

The long-range paths of length higher than 8 were compared in Dri_DNA_ and Dri_FREE_ simulations and the differences between them mapped on the 3D structure (**Figure 5** **upper panel**). We noticed that the presence of DNA promotes a larger number of long-range paths to L2 and L1. Among those paths, Y119 (Y109 in Dri) plays a crucial role being involved in one of the major route of communication to the DNA-binding loops, along with L116 (L106 in Dri). In fact, they belong to the paths from K113 to both L56 at the base of L1 and L87-I91-P88 in L2 (**Figure 5** **, lower panel**). In Dri_FREE_, some of the paths of length higher than 8 and that link the C-terminal helix or its surroundings to other protein regions distal to the DNA-binding sites are instead lost in Dri_DNA_ simulations (blue lines in **Figure 5** **, lower panel**). These data further enforce the notion that the presence of DNA promotes a more channeled communication toward the DNA-binding sites.

**Figure 6:**
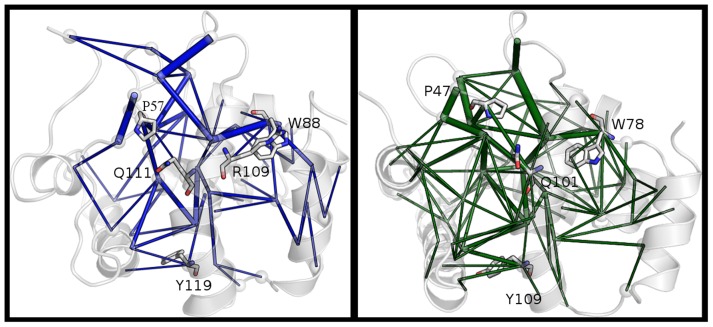
Shortest paths of long-range communication in ARID3A and Dri. The edges which are present with highest probability in the paths of long-range communication identified by PSN-LMI approach are indicated for ARID3A_FREE_ (blue) and Dri_FREE_ (dark green) simulations by lines of thickness proportional to the occurrence probability. The residues connected by the edges are indicated as spheres, whereas the residues known to compromise the DNA-binding capabilities in ARID3A, along with Q111/Q101 and R109/R99 are shown as sticks if identified in the graph.

For each system, all the paths to L1 and L2 identified above were joined in one single graph. Then, the nodes belonging to this graph were connected by edges whose thickness is proportional to the probability to find in the graph the same connection in different communication paths, providing the final graphs reported in **Figure 6** for ARID3A and Dri. The analysis provides an overview of the residues and the connections that are more represented in the paths from distal sites of ARID domains to the DNA-binding loops.

We found that the residues that were experimentally investigated [9] and for which alanine mutations have a direct or indirect effects on DNA-binding capabilities (i.e. Y119, P57 and W88) are highly represented in the paths of communication, as well as R109 and Q111. There are also other residues interested by highly abundant edges as M59, L66, L108 and L116 in suitable positions to be mediator of the communication. Most of these residues are located in helix α5. M59A, L66A, L108A and L116A mutations are also predicted as destabilizing the 3D structure (ΔΔG values in the range of 3.24–4.17 kcal/mol) by Fold-X, as the W88A mutation discussed above. Thus, they are likely to be not only important residues for long-range communication but also in maintaining the correct 3D architecture.

In summary, Y119 and Q111 are suggested as important hubs for structural communication within the ARID domains. They can be modulated by DNA-interaction and are also among the most represented nodes in the long-range paths from protein distal sites to the DNA-binding loops, and alanine mutations of these residues are also predicted not to affect the protein stability. They are thus suitable candidate to verify their role as important mediators of communication to the DNA-binding loops.

### Y119 and Q111 are crucial residues for long-range communication to the DNA-binding interface

Experimental mutagenesis pointed out that Y119A mutation can affect DNA-binding capability of ARID3A [9]. Y119 is not in direct contact with the DNA molecule since it is more than 20 Å far from the DNA-binding interface and partially solvent exposed (average solvent accessibility of the side chain in the simulations higher than 15%). It has therefore to exert its effect long-range. It thus represents a good candidate to further investigate the communication to the DNA-binding loops, as well as to probe if the paths identified by the PSN/LMI approaches can modulate long-range the conformation and dynamics of the DNA-binding loops. In particular, we compared the wild type MD simulations of ARID3A and Dri with simulations of Y119A/Y109A mutants (ARID3A_Y119A_, Dri_FREE-Y109A_ and Dri_DNA-Y109A_) with the same approaches described in the previous Sections. We also include mutations in Q111/Q101, which can also be a mediator of long-range communication to the DNA interface and for which mutations to Asn or Ala are predicted to have neutral effects on protein stability by FoldX. In particular, we included Q111N mutation as a control in our simulations since it is a conservative mutation that we did not expect to affect the overall dynamics. Moreover, since the only relevant difference between Asn and Gln residues is the side-chain length, we can also use this mutant to verify if even subtle changes in the side chains of crucial nodes for structural communication can affect the communication paths.

**Figure 7:**
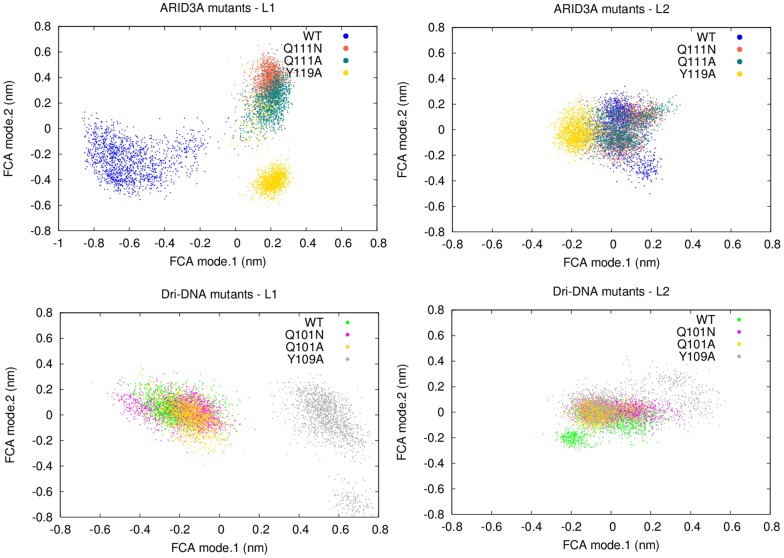
FCA modes of L1 and L2 in wt ARID3A_FREE_ or Dri_DNA_ and their mutants. **Upper panels**) The two panels show the projection along FCA mode 1 and FCA mode 2 for L1 and L2 in ARID3A_FREE_ and its mutants. ARID3A_FREE_, ARID3A_Y119A_, ARID3A_Q111A_ and ARID3A_Q111N_ are shown with different shade of colors. **Bottom panels**) The two panels show the projection along FCA mode 1 and FCA mode 2 for L1 and L2 in Dri_DNA_ and its mutants. Dri_DNA_, Dri_Y119A_, Dri_Q111A_ and Dri_Q111N_ are shown with different shade of colors. The projections report one replicate of each system, for sake of clarity. The same analysis was carried out for each combination of replicates of wild type and mutant variants and it provides similar results, as also expected by the high overlap in the essential subspace between individual replicates of the same system (root mean square inner product larger than 0.8).

We thus compare wild-type ARID domain dynamics to the different mutant variants by Full Correlation Analysis (FCA) analysis of L1 and L2 loops in a common reference subspace. FCA analysis of the L1 and L2 loops only shows that those mutations affect the dynamic properties of the DNA-binding loops when compared to the wt (**Figure 7** **upper panels**). In particular, Y119A has the most prominent effects on the native dynamics of both the DNA-binding loops, whereas Q111A and Q111N have a major effect mainly on L1 and more native-like patterns for L2.

It can be argued that the effects induced upon these mutations are less detrimental if DNA is present. Therefore, to further verify that the effects induced by those mutations can be identified also in DNA-bound form, we carried out also MD simulations of Dri_DNA_ mutant variants at the position corresponding to the ARID3A mutation sites (i.e. Q101A, Q101N and Y109A). The FCA analysis of L1 and L2 was carried out also for wild-type Dri_DNA_ and its mutant variant confirming the picture described above for Y119A mutant (**Figure 7** **bottom panels**). On the contrary, mutations at the 101 site in Dri (Q111 in ARID3A) feature less detrimental effects on the native dynamics in presence of the DNA. This result suggests that the DNA can partially rescue the structural effects induced by Q111 mutations.

**Figure 8:**
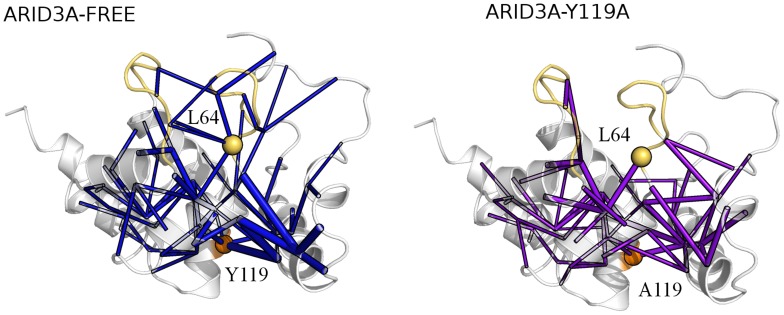
Paths of long-range communication mediated by Y119 in wt and Y119A ARID3A. The shortest paths of communications mediated by Y119 in ARID3A_FREE_ (left panel) and ARID3A_Y119A_ (right panel) simulations as identified by the PSN-LMI approach are shown as cylinders of thickness proportional to the probability of occurrence. The Y119 Cα and the L64 Cα are also indicated by orange and yellow spheres, respectively. L1 and L2 are highlighted in yellow.

**Figure 9:**
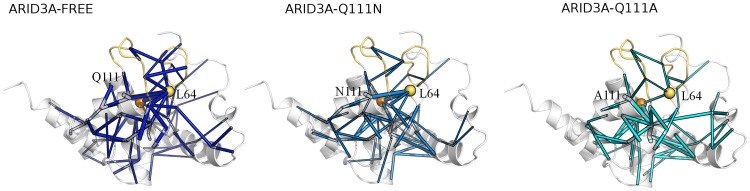
Paths of long-range communication mediated by Q111 in wt, Q111A and Q111N ARID3A. The shortest paths of communications mediated by Q111 in ARID3A_FREE_, (left panel) ARID3A_Q111A_ (middle panel) and ARID3A_Q111N_ (right panel) simulations as identified by the PSN-LMI approach are shown as cylinders of thickness proportional to the probability of occurrence. The Q111 Cα and the L64 Cα are also indicated by orange and yellow spheres, respectively. L1 and L2 are highlighted in yellow.

To investigate the role of those residues in the communication routes to the DNA-binding loops, all the shortest paths with occurrence probability higher than 15% identified by PSN-LMI, which starts by Y119 (**Figure 8** **left panel**) or Q111 (**Figure 9** **left panel**) and are directed toward other residues are considered with respect to their location on the 3D structure of ARID3A_FREE_ and compared to the ones identified for the same residues in the mutants Y119A (**Figure 8** **right panel**), Q111N (**Figure 9** **middle panel**) and Q111A (**Figure 9** **right panel**).

In the wt dynamics, it turns out that part of the paths from Y119 and Q111 are directed toward L1 and L2 and other regions close to the interface for DNA interaction, including the highly interconnected intermediate node L64 (showed as a yellow sphere in **Figures 8** **and** **9**).

Alanine mutations at both 119 and 111 sites dramatically affect the communication in ARID3A. Indeed, the mutations perturb the long-range paths, especially the ones of length greater than 7. They either decrease the probability of occurrence of the paths or they weaken the communication so that the paths are preserved but shorter in length and they cannot successfully reach the DNA-binding loops. Moreover, there are cases in which the mutations cause a major perturbation in the native paths, re-directing the communication toward other regions of the protein (**Figure 8** **and** **9**) and in particular affecting L1.

The Q111N mutation is more similar to the wild type (**Figure 9** **middle panel**). Nevertheless, even the subtle replacement of the Gln with a shorter side-chain residue (as Asn) decreases the probability of occurrence of some of the paths directed to the DNA-binding loops. The mutation indeed causes a weakening of the communication to L1.

## Discussion

Methods inspired to graph theory are widely used to study protein structure-function relationships [13]–[15], [17]–[19], [24]–[25] and they have also been applied to the study of complex biological phenomena such as long-range intra- and intermolecular communication [13]–[15], [17]–[19], [24]–[32].

Here, we integrated graph theory and MD simulations to describe the structural dynamics and intramolecular communication in the ARID family of DNA-binding domains, which have been so far poorly structurally characterized. Our simulated ensembles were also first evaluated for consistency with the available experimental information from NMR. The crucial cutoffs for PSN analyses have been evaluated comparing different replicates for each system, along with simulations of different lengths. We have then examined how dynamical properties of ARID domains are influenced by the interaction with DNA or by mutations at critical sites in the communication paths to the DNA-binding loops.

We are aware that our approach is mainly protein-centered, even if simulations are carried out in explicit solvent and thus the dynamics we are describing and the related paths are ultimately influenced by the solvent dynamics too. A network description of the clusters of water molecules around the protein surface or in protein cavities, as recently investigated by other techniques [33]–[36] may complement the PSN information and it can be consider for future applications.

The PSN/MD approaches here employed provide also a global description of the dynamical communication within the ARID domain, which might be difficult to obtain by other means. More generally, we thus hope that these approaches can be an useful starting point in cases where little experimental information is available to guide further experimental characterization. The definition of the nodes and their edges that more frequently populate the paths of long-range communication in the PSN/LMI approach can also be a complementary tool for the identification of important residues in the dynamic networks, i.e. they for example complement the information from hub detection in PSN. This technique can be employed to identify hot-spot residues for protein function and stability, as we here showed integrating them with Fold-X calculations of mutations in the hubs. Indeed, the edges with high occurrence probability in the communication paths have the potential to act as fundamental signal transmitters to allow the information flow throughout the protein structure.

On the biological side, our results show that structural communication in ARID domains can pass through a subset of conserved hubs, among which Y119 and W88 are found. Y119 and W88 were also experimentally investigated in ARID3A and are known to affect the protein function and interaction with DNA [9]. Other relevant residues to provide the native communication flow are suggested to be Q111, L116, L108, L66 and M59. We also evaluated in our MD framework Y119A, Q111A and Q111N mutations that turned out to affect the communication routes of the native protein to the DNA-binding loops at different extent. Most of those residues are located in the helix α5 that we thus found to be a central region for the long-range communication to the DNA binding loops.

In our MD ensembles, pre-existing communication paths in the DNA-unbound states are directed toward L1 and L2 at the DNA-binding interface in the free proteins and they are strengthened by the interaction with DNA. Y119, Q111 and the other residues mentioned above turned out to be critical nodes for the long-range communication to the DNA-binding loop.

Interestingly, the region including Y119 and its surrounding (F38, F67, M68, Y70, V71, L72 and T74) is predicted as a hotspot for protein-protein interaction by InterProtSurf [37]. Our results can thus boost future research in the field of ARID domains to characterize protein-protein interaction mapping at this region and modulated by DNA-binding.

It appears that the ARID domains may exist as an ensemble of substates in solution, which can be shifted by external perturbation, such as the interaction with DNA in our study. L1 and L2 DNA-binding loops play an important role in determining the conformational changes between the different ARID substates and their dynamical properties are directly influenced by DNA interaction, but the effect can also be transmitted long-range by intramolecular paths, which have their heart in the helix α5.

## Materials and Methods

### Starting structures for MD simulations

The known NMR structures of human ARID3A (ARID3A_FREE_, PDB entry 2KK0, [6]) and *Drosophila melanogaster* Dri (Dri_FREE_ PDB entry 1C20, [8]) domains free in solution were used as starting structures for MD simulations (**Table 1**). In particular, from the PDB entry 2KK0 only the atomic coordinates referred to the ARID3A protein were considered, excluding the N-terminal His-tag construct and the residues belonging to the disordered N-terminal tail. Several 100-ns independent simulations of ARID3A_FREE_ (four replicates) and Dri_FREE_ (six replicates) were carried out using as initial structure the first conformer observed by NMR spectroscopy. One of the DriFREE simulations was extended to 1 µs. Simulations (four replicates) were also carried out for Dri in complex with the DNA (Dri_DNA_), starting from the first NMR conformer in the PDB entry 1KQQ [7]) (**Table 1**). The availability of independent simulations of the two homologous proteins and over different timescales allowed a better assessment of the reproducibility of the results and the robustness of the PSN-MD approach. Two replicates for each mutant (Y119A, Q111A and Q111N) ARID3A and Dri variants, with and without DNA, were also carried out upon *in-silico* mutagenesis with Pymol (www.pymol.org). Unfolding simulations at 500 K of 100 ns were also performed for both Dri_FREE_ and ARID3A_FREE_ to employ as a control in the evaluation of the correlated motions.

### Molecular dynamics (MD) simulations

Explicit solvent MD simulations were performed using the 4.5.3 version of the GROMACS software [38] with the CHARMM22* force field [11]. The initial structures were embedded in a dodecahedral box of TIP3P water molecules [39]. Periodic boundary conditions were employed. All the protein atoms were at a distance equal or greater than 1.0 nm from the box edges. To neutralize the overall charge of the system, a number of water molecules equal to the protein net charge were replaced by counter-ions.

Each system was initially relaxed by 10000 steps of energy minimization by the steepest descent method. The optimization step was followed by 50 ps of solvent equilibration at 300K, while restraining the protein atomic positions using a harmonic potential. Each system was then slowly equilibrated to the target temperature (300 K) and pressure (1 bar) through thermalization and a series of pressurization simulations of 100 ps each.

Productive MD simulations were performed in the isothermal-isobaric (NPT) ensemble at 300K and 1 bar, using an external Berendsen bath with thermal and pressure coupling of 0.1 and 1 ps respectively. The LINCS algorithm [40] was used to constrain heavy-atom bonds, allowing for a 2 fs time-step. Long-range electrostatic interactions were calculated using the Particle-Mesh Ewald (PME) summation scheme [41]. Van der Waals and short-range Coulomb interactions were truncated at 0.9 nm. The non-bonded pair list was updated every 10 steps and conformations were stored every 4 ps.

The main chain root mean square deviation (rmsd), which is a parameter used to evaluate the stability of MD trajectories, was computed using the corresponding NMR structure as a reference. The first 10 ns of each trajectory were discarded as initial equilibration for each simulation. Indeed, upon 10 ns the trajectories were generally characterized by average main chain rmsd lower than 0.29±0.07 nm.

### Full Correlation Analysis (FCA)

FCA is based on the calculation of Mutual Information (MI), which quantifies any kind of correlations including linear, non-linear or higher-order contributions. It has been showed that FCA lead to better-resolved conformational substates or modes than classical Principal Component Analysis (PCA) [42]–[44] and that these are more often aligned with the actual transition pathways in the structural ensembles [45] Here, the FCA analyses were carried out for the Cα atoms only and using the first 25 eigenvectors from Cα PCA, as suggested in ref. [45].

### Linear Mutual Information matrices (LMI)

LMI was employed to quantify correlated motions from MD simulations since it has the advantage of not depending on the relative orientations of the fluctuations [16], making it possible to identify correlated motions unregard of the difference between their orientations in space. LMI can range from 0 (uncorrelated motions) to 1 (fully correlated motions). LMI matrices including the correlated motion between pairs of residues were obtained computing Cα LMI using non over-lapping averaging windows of five ns (**Figure S3**). A cutoff of 0.5 was selected to reduce noise and to identify significant correlations, aiming to exclude from the analyses the pairs of residues that are poorly communicating with each other and likely to be characterized by almost uncoupled motions. To identify a suitable cutoff for significant correlation, differences between average LMI matrices calculated with one-ns and five-ns averaging windows for the same MD run, as well as between average LMI matrices calculated for the different replicates of the same protein (i.e. different force-field descriptions or different starting structures) were calculated. The probability density function for the difference values was then calculated, along with the maximum value of the difference and the pairs of residues, which were interested by the highest differences for each protein. In particular, no differences were identified higher than 0.35 and related to just few pairs involving the C- and N-terminal residues.

Moreover, the Forbenius norm between the different LMI matrices have been calculated to quantify the similarity between the LMI matrices from different replicates of the same system.

In particular, given two matrices of the same size, it is possible to evaluate their degree of similarity by calculating the Frobenius norm of the difference between them. The Frobenius norm for two given LMI square matrices of order *m*, LMI_A_ and LMI_B_, was calculated as follows
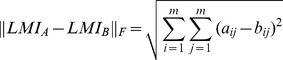
where *a_ij_* and *b_ij_* are elements of respectively LMI_A_ and LMI_B_ and the matrix order *m* is equal to the number of residues of the target protein.

### Protein Structure Network (PSN) and shortest correlated path of communication

The PSN approach was integrated to the LMI matrices of correlated motions (PSN/LMI) [13]–[15] to identify the most relevant communication paths in ARID3A and Dri simulations. The PSN method employs the graph formalism to define a network of interacting residues in a given protein from the number of non-covalent interacting atoms, using a calculated *I_ij_* interaction strength value as the edge weight, where *i* and *j* are residue identifiers. This value is calculated on the basis of the number of distinct atom pairs (*n_ij_*) between residues *i* and *j* within a distance cutoff of 0.45 nm
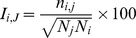
where N_i_ and N_j_ are normalization values for residues *i* and *j* obtained from a statistically significant protein dataset [17], [46]. Nodes are connected to edges when *I_ij_*>*I_min_*, where I_min_ is a defined cutoff value.

The residues that have zero edges are termed as orphans, whereas those that are involved in more than four edges are referred as hubs at a specific I_min_ value. The node inter-connectivity is used to highlight the cluster-forming nodes, where a cluster is a set of connected residues in the graph. The node clustering procedure is such that nodes were iteratively assigned to a cluster if they could establish a link with at least one node in the cluster. A node not linkable to existing clusters initiates a new cluster in an iterative procedure until the node list is completed. Cluster size, defined as the number of nodes, is known to vary as a function of the selected I_min_ and the size of the largest cluster is used to calculate the I_crit_ value. At I_min_ = I_crit_, the weak node interactions are generally discarded. Therefore, in our calculation I_min_ was set equal to I_crit_, where I_crit_ is the value of I_min_ at which the size of the largest clusters in the graph significantly changes [17], [46]. In particular, an I_crit_ value of 7 was obtained for all the MD runs under investigation.

To obtain a single PSN for each MD trajectory, a PSN was calculated for each frame and only edges present in at least half of the simulation frames were considered. For each pair of nodes in the PSN graph, the Floyd-Warshall algorithm was employed to identify the shortest path of communication. The distance between directly connected residues in the graph was considered to be 1, and the shortest path was identified as the path in which the two residues were non-covalently connected by the smallest number of intermediate nodes. Only the shortest paths in which at least one identified node featured a significant correlation value (0.5) with one of the residues of the select pair were retained. The correlation values were evaluated by the LMI analyses described above. All the PSN, LMI and PSN-LMI calculations were performed using the WORDOM MD trajectories analysis suite [23] and in-house available Python scripts for analyses of WORDOM outputs. The plot of the paths on the 3D structures were carried out using the xPyder [47] plugin for PyMOL.

### Fold-X calculations

To predict the effects induced by ARID3A mutations on protein stability, we used the ARID3A NMR structure (first conformer in the PDB entry 2KK0) that was repaired by the Repair module of Fold-X. To assess the effects of the mutations, we then use the BuildModel module of Fold-X v.3.0 [22] and we carried out 5 independent run for each mutations.

## Supporting Information

Figure S1 **ClustalW alignment between Arid3A and Dri.** ‘*’, ‘:’ and ‘.’ indicate identical, strictly similar and similar residues, respectively. Residues for which mutations, described in ref. [9], are known to affect the DNA-binding properties are highlighted in yellow.
(DOCX)

Figure S2 **Chemical shifts prediction for 1 µs Dri_FREE_ simulation.** Chemical shifts were predicted from the 1 µs Dri_FREE_ simulation by the PPM webserver [12] and compared to experimental chemical shifts of 1C20 PDB entry. Rmsd values for the different atom types (Cα, Cβ, C′, HN and N) have been plotted as a function of the simulation time. The green dotted line corresponds to the rmsd value calculated by PPM for the starting structure of the simulation (first conformer in 1C20 PDB entry).
(DOCX)

Figure S3 **LMI matrices describing correlated motions. Average LMI matrices achieved with five-ns time-windows.** Examples with simulations of different length (i.e 100 ns (A–C) and 1 µs(D)) and of different replicate of the same system (i.e. r1 (A), r5 (B) and r6 (C)) for Dri_FREE_ simulations. The LMI matrices are overall very similar, showing a robust description of the correlated motions upon averaging over five ns in the target proteins.
(DOCX)

Figure S4 **Hub residues of ARID domains.** The connectivity degree for each PSN hub of ARID3A_FREE_ (A) and 1 µs Dri_FREE_ (B) simulations are shown as a function of the protein residue. Since in a PSN a hub is defined as a residue connected by at least three edges, all the residues with node degree lower than three are set at zero.
(DOCX)

Figure S5 **Location of the residues known to affect DNA-binding capabilities in ARID3A if mutated to alanine [9].** Y119 is show as sticks and dots, whereas P57, W88 and F106 are shown as sticks.
(DOCX)
