# Supplementary material for: Communication Routes in ARID Domains between Distal Residues in Helix 5 and the DNA-Binding Loops
Source: PLoS Comput Biol. 2014 Sep 4;10(9):e1003744. doi: 10.1371/journal.pcbi.1003744 (PMC4154638; doi:10.1371/journal.pcbi.1003744)
Supplement: Figure S1 — ClustalW alignment between Arid3A and Dri. ‘*’, ‘:’ and ‘.’ indicate identical, strictly similar and similar residues, respectively. Residues for which mutations, described in ref. [9], are known to affect the DNA-binding properties are highlighted in yellow. (DOCX) [file pcbi.1003744.s001.docx]

**Figure S1. ClustalW alignment between ARID3A and Dri.** ‘*’, ‘:’ and ‘.’ indicate identical, strictly similar and similar residues, respectively. Residues for which mutations, described in ref. [9] are known to affect the DNA-binding properties are highlighted in yellow.

ARID3A PDHGDWTYEEQFK---QLYELDGDPKRKEFLDDLFSFMQKRGTPVNRIPIMAKQVLDLFM

Dri QNNG-WSFEEQFKQVRQLYEINDDPKRKEFLDDLFSFMQKRGTPINRLPIMAKSVLDLYE

:.**::***** ****::.*********************:**:*****.****:

ARID3A LYVLVTEKGGLVEVINKKLWREITKGLNLPTSITSAAFTLRTQYMKYLYPYECEKRGLSN

Dri LYNLVIARGGLVDVINKKLWQEIIKGLHLPSSITSAAFTLRTQYMKYLYPYECEKKNLST

** ** :****:*******:** ***:**:************************:.**.

ARID3A PNELQAAIDSNR

Dri PAELQAAIDGNR

* *******.**
