# Supplementary material for: Communication Routes in ARID Domains between Distal Residues in Helix 5 and the DNA-Binding Loops
Source: PLoS Comput Biol. 2014 Sep 4;10(9):e1003744. doi: 10.1371/journal.pcbi.1003744 (PMC4154638; doi:10.1371/journal.pcbi.1003744)
Supplement: Figure S2 — Chemical shifts prediction for 1 µs DriFREE simulation. Chemical shifts were predicted from the 1 µs DriFREE simulation by the PPM webserver [12] and compared to experimental chemical shifts of 1C20 PDB entry. Rmsd values for the different atom types (Cα, Cβ, C′, HN and N) have been plotted as a function of the simulation time. The green dotted line corresponds to the rmsd value calculated by PPM for the starting structure of the simulation (first conformer in 1C20 PDB entry). (DOCX) [file pcbi.1003744.s002.docx]

**Figure S2. Chemical shifts prediction for 1** µs Dri_FREE_ **simulation.** Chemical shifts were predicted from the 1 µs Dri_FREE_ simulation by the PPM webserver [12] and compared to experimental chemical shifts of 1C20 PDB entry. Rmsd values for the different atom types (Cα, Cβ, C’, HN and N) have been plotted as a function of the simulation time. The green dotted line corresponds to the rmsd value calculated by PPM for the starting structure of the simulation (first conformer in 1C20 PDB entry).

**
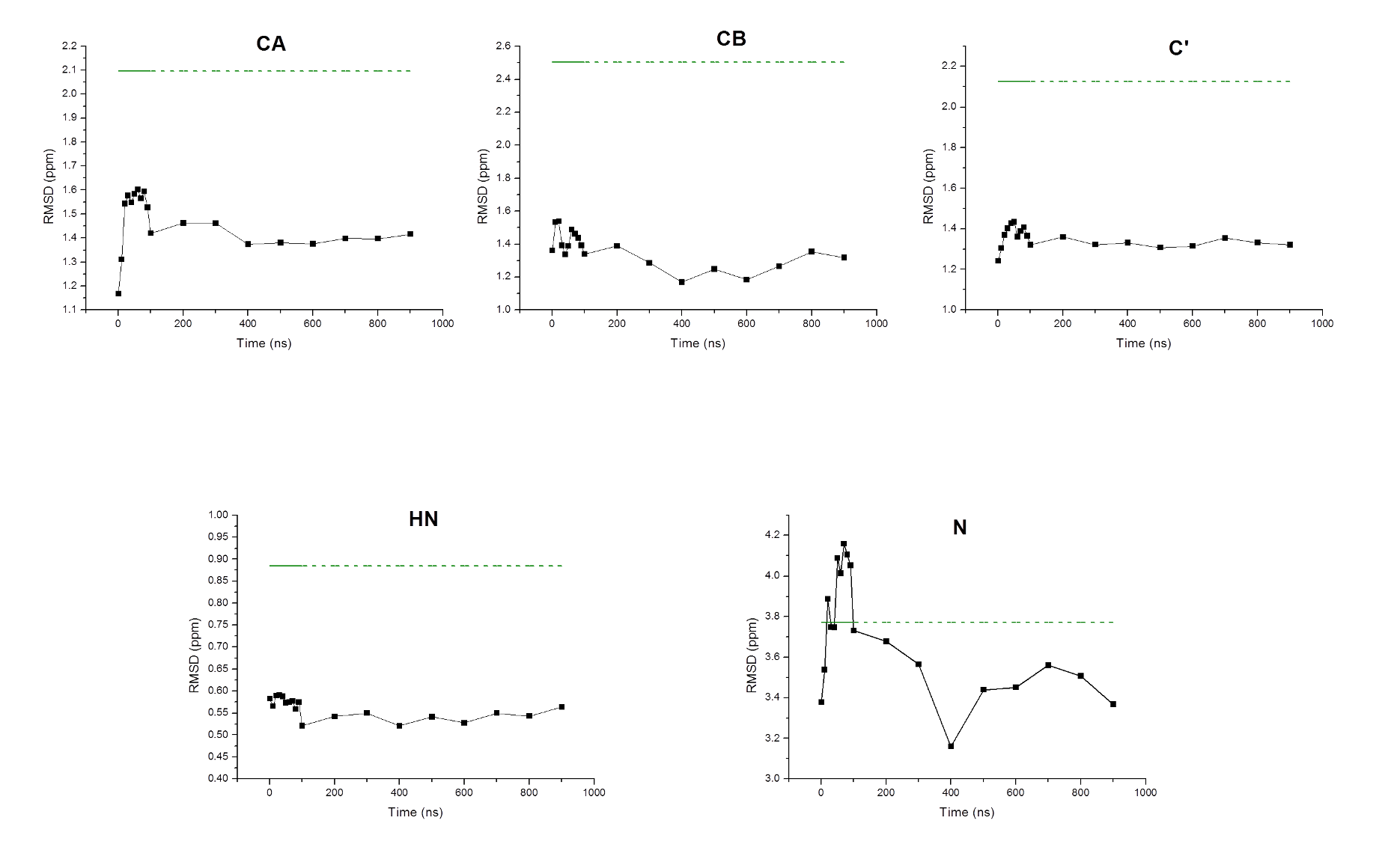
**
