# Supplementary material for: Communication Routes in ARID Domains between Distal Residues in Helix 5 and the DNA-Binding Loops
Source: PLoS Comput Biol. 2014 Sep 4;10(9):e1003744. doi: 10.1371/journal.pcbi.1003744 (PMC4154638; doi:10.1371/journal.pcbi.1003744)
Supplement: Figure S3 — LMI matrices describing correlated motions. Average LMI matrices achieved with five-ns time-windows. Examples with simulations of different length (i.e 100 ns (A–C) and 1 µs(D)) and of different replicate of the same system (i.e. r1 (A), r5 (B) and r6 (C)) for DriFREE simulations. The LMI matrices are overall very similar, showing a robust description of the correlated motions upon averaging over five ns in the target proteins. (DOCX) [file pcbi.1003744.s003.docx]

**Figure S3. LMI matrices describing correlated motions. Average LMI matrices achieved with 5ns time-windows.** Examples with simulations of different lengths (i.e 100 ns (A-C) and one μs(D)) and of different replicates of the same system (i.e. r1 (A), r5 (B) and r6 (C)) for Dri_FREE_ simulations. The LMI matrices are overall very similar, showing a robust description of the correlated motions upon averaging over five ns in the target proteins.

D

C

B

A
