# Supplementary material for: Communication Routes in ARID Domains between Distal Residues in Helix 5 and the DNA-Binding Loops
Source: PLoS Comput Biol. 2014 Sep 4;10(9):e1003744. doi: 10.1371/journal.pcbi.1003744 (PMC4154638; doi:10.1371/journal.pcbi.1003744)
Supplement: Figure S4 — Hub residues of ARID domains. The connectivity degree for each PSN hub of ARID3AFREE (A) and 1 µs DriFREE (B) simulations are shown as a function of the protein residue. Since in a PSN a hub is defined as a residue connected by at least three edges, all the residues with node degree lower than three are set at zero. (DOCX) [file pcbi.1003744.s004.docx]

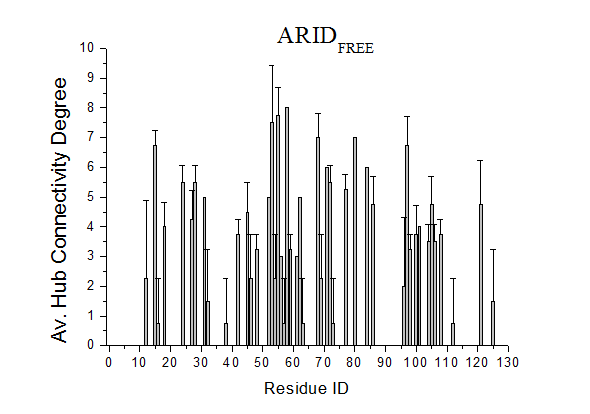
**Figure S4. Hub residues of ARID domains.** The connectivity degree for each PSN hub of ARID3A_FREE_ (A) and 1 µs Dri_FREE_ (B) simulations are shown as a function of the protein residue. Since in a PSN a hub is defined as a residue connected by at least three edges, all the residues with node degree lower than three are set at zero.

A


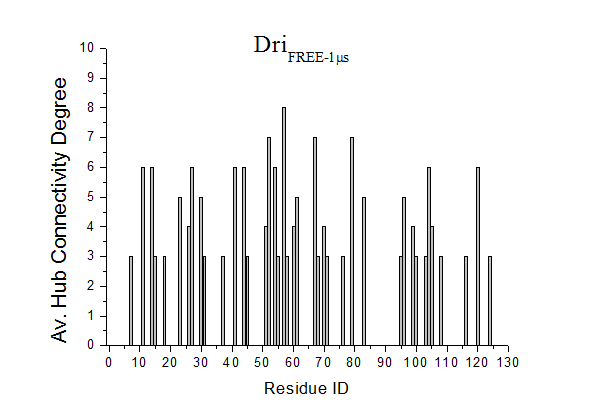


B
