# Supplementary material for: Communication Routes in ARID Domains between Distal Residues in Helix 5 and the DNA-Binding Loops
Source: PLoS Comput Biol. 2014 Sep 4;10(9):e1003744. doi: 10.1371/journal.pcbi.1003744 (PMC4154638; doi:10.1371/journal.pcbi.1003744)
Supplement: Figure S5 — Location of the residues known to affect DNA-binding capabilities in ARID3A if mutated to alanine [9]. Y119 is show as sticks and dots, whereas P57, W88 and F106 are shown as sticks. (DOCX) [file pcbi.1003744.s005.docx]

**Figure S5. Location of the residues known to affect DNA-binding capabilities in ARID3A if mutated to alanine** **[9].** Y119 is show as sticks and dots, whereas P57, W88 and F106 are shown as sticks.

**
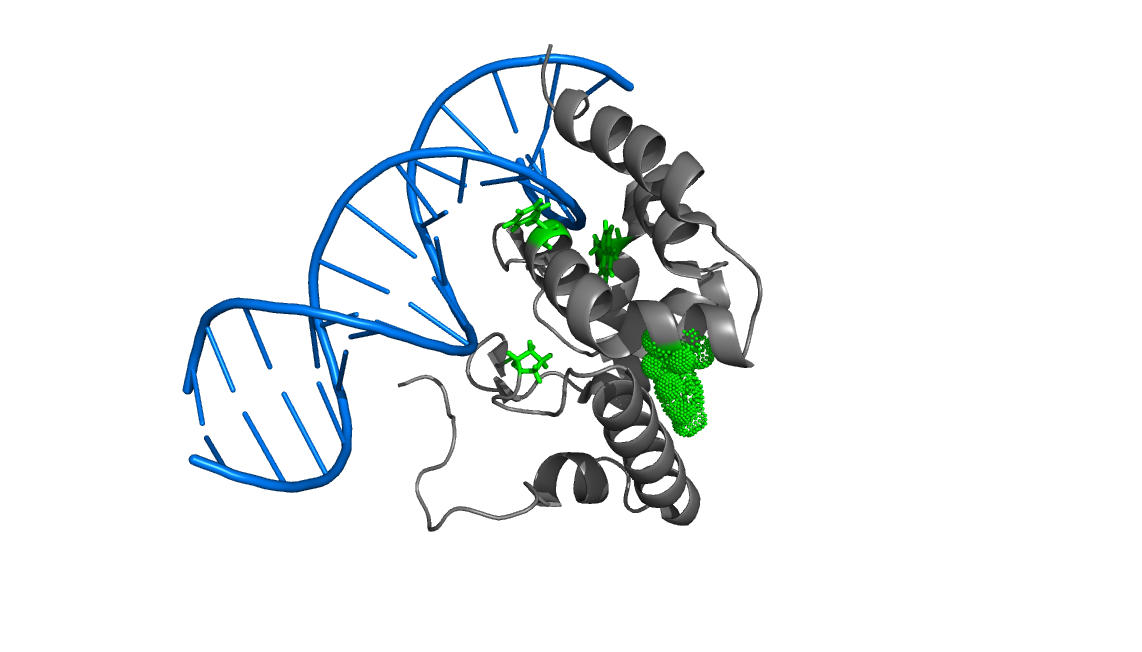
**

**W88**

**F106**

**P57**

**Y119**
